# Preventive Effect and Mechanism of *Crossostephium chinense* Extract on Balloon Angioplasty-Induced Neointimal Hyperplasia

**DOI:** 10.1155/2021/8466543

**Published:** 2021-06-30

**Authors:** Chun-Hsu Pan, Yu-Pei Lin, Jie-Yu Wang, Hui-Yu Huang, Shun-Cheng Huang, Ji-Mehng Lo, Chieh-Hsi Wu

**Affiliations:** ^1^Ph.D. Program in Drug Discovery and Development Industry, College of Pharmacy, Taipei Medical University, Taipei 11031, Taiwan; ^2^School of Pharmacy, Taipei Medical University, Taipei 11031, Taiwan; ^3^Division of Endocrinology and Metabolism, Department of Internal Medicine, Taipei Medical University Hospital, Taipei 11031, Taiwan; ^4^Graduate Institute of Metabolism and Obesity Sciences, Taipei Medical University, Taipei 11031, Taiwan; ^5^Biomedical Technology and Device Research Laboratories, Industrial Technology Research Institute, Hsinchu 31040, Taiwan

## Abstract

Balloon angioplasty-induced neointimal hyperplasia remains a clinical problem that must be resolved. The bioactivities of the *Crossostephium chinense* extract (CCE) have demonstrated potential in preventing the progression of restenosis. The present study evaluated whether CCE can suppress balloon angioplasty-induced neointima formation and elucidated its possible pharmacological mechanisms. A rat model of carotid arterial balloon angioplasty was established to evaluate the inhibitory effect of CCEs on neointimal hyperplasia. Two cell lines, A10 vascular smooth muscle cells (VSMCs) and RAW264.7 macrophages, were used to investigate the potential regulatory activities and pharmacological mechanisms of CCEs in cell proliferation and migration and in inflammation. Our in vitro results indicated that CCE3, the ethanolic extract of *C. chinense*, exerted the strongest growth inhibitory and antimigratory effects on VSMCs. CCE3 blocked the activation of focal adhesion kinase, platelet-derived growth factor receptor-*β* (PDGFRB), and its downstream molecules (AKT and mTOR) and reduced the expression of matrix metalloproteinase-2. In addition, our findings revealed that CCE3 significantly increased the expression of miRNA-132, an inhibitory regulator of inflammation and restenosis, and suppressed the expression of inflammation-related molecules (inducible nitric oxide synthase, cyclooxygenase-2, interleukin- (IL-) 1*β*, and IL-6). Our in vivo study results indicated that balloon injury-induced neointimal hyperplasia was inhibited by CCE3. CCE3 could reduce neointima formation in balloon-injured arteries, and this effect may be partially attributed to the CCE3-induced suppression of PDGFRB-mediated downstream pathways and inflammation-related molecules.

## 1. Introduction

Percutaneous coronary intervention is a common surgical procedure used to reopen an acute arterial obstruction by inserting a balloon catheter in occlusive sites, followed by intraluminal inflation. However, subsequent restenosis (or neointimal hyperplasia) was observed to develop in approximately 20–50% of patients receiving balloon angioplasty or vascular stent implantation [1, 2]. Abnormal proliferation and migration of vascular smooth muscle cells (VSMCs) have been verified to play key pathological roles in the progression of neointimal hyperplasia; thus, VSMCs have become a potential therapeutic target for restenosis induced by vascular drug-eluting stents [3, 4].

Some regulatory factors, such as platelet-derived growth factor (PDGF) and inflammation, play crucial pathological roles in VSMCs during neointima formation. PDGF directly regulates and promotes the proliferation and migration of VSMCs through PDGF receptors (PDGFRs), especially PDGFR-*β* (PDGFRB), and stimulates neointima thickening [5–7]. Blocking PDGF by using neutralizing antibodies or receptor blockers could effectively reduce neointima formation in animal models [8–10]. In addition, PDGF can upregulate the expression of monocyte chemoattractant protein-1 (MCP-1) to recruit macrophages and monocytes to injured tissues for participation in local inflammation [11, 12]. Moreover, inflammatory cytokines, such as interleukin-1*β* (IL-1*β*) and IL-6, released from recruited immunocytes can exacerbate VSMC proliferation and migration and neointimal hyperplasia [13, 14], and therapeutic interventions that involve the suppression of inflammation can alleviate neointima formation [15, 16]. The aforementioned studies have provided evidence for the pathological roles of PDGF and inflammation in the progression of restenosis and have indicated the complex relationship between restenosis and these pathological factors.

Natural-derived products have been comprehensively used in the discovery of new drugs. *Crossostephium chinense* (also called Hai-Fu-Rung) is a Chinese herbal medicine that is traditionally used to manage arthritis and rheumatism [17]. *C. chinense* extract (CCE) has beneficial effects; for example, it inhibits the growth and migration of human cancer cells [4, 18, 19] and reduces inflammation [4, 20], suggesting that CCE can be used to prevent neointima formation. The present study evaluated whether CCE can inhibit balloon angioplasty-induced neointimal formation and elucidated the possible pharmacological mechanisms underlying how CCE regulates VSMCs.

## 2. Materials and Methods

### 2.1. Chemicals and Reagents

Primary antibodies against focal adhesion kinase (FAK; #3285s), phospho-FAK (#8556s), and the mechanistic target of rapamycin (mTOR; #2972s) were purchased from Cell Signaling Technology (Beverly, MA, USA). Primary antibodies against AKT (#GTX121937), phospho-AKT (#ab28821), PDGFRB (#GTX61115), and phospho-PDGFRB (#GTX61797) were purchased from GeneTex (Irvine, CA, USA). Primary antibodies against cyclooxygenase-2 (COX-2; #160126), inducible nitric oxide synthase (iNOS; #610432), glyceraldehyde 3-phosphate dehydrogenase (GAPDH; #NB300-221), and phospho-mTOR (#ab109268) were purchased from Cayman Chemical (Ann Arbor, MI, USA), BD Transduction Laboratories (Lexington, KY, USA), Novus Biologicals (Centennial, CO, USA), and Abcam (Cambridge, MA, USA), respectively. Horseradish peroxidase- (HRP-) conjugated secondary antibodies against mouse (#GTX213112-01) and rabbit (#GTX213110-01) immunoglobulin G were also purchased from GeneTex. All other reagents that are not listed were purchased from Sigma-Aldrich (Louis, MO, USA).

### 2.2. Preparation of CCE

*C. chinense* was harvested from Matsu islands in Lienchiang County, Taiwan. The plant's leaves and stems were collected, cut into pieces, and steam distilled to obtain an aqueous solution. The aqueous portion was freeze-dried to obtain the initial water extract (CCE1). Subsequently, the residue was sequentially refluxed using n-hexane and ethanol for 8 h to obtain the second extract (CCE2) and third extract (CCE3). The organic solvent extracts were concentrated under reduced pressure at 60°C.

### 2.3. High-Performance Liquid Chromatography

High-performance liquid chromatography (HPLC) was performed following the procedure reported in our previous study with a minor modification [21]. In brief, the CCEs were dissolved in HPLC-grade methanol and passed through a 0.2-*μ*m filter; then, 20 *μ*L of the solution was loaded into an HPLC column. Reverse-phase HPLC was run using an Inertsil ODS-3V C18 HPLC-cartridge (5 *μ*m particle size, 250 × 4.6 mm; GL Sciences, Tokyo, Japan) at a flow rate of 1.0 mL/min at 25°C; a detection wavelength of 220 nm was used. Two mobile phases, namely, methanol and 0.1% phosphoric acid aqueous solution, were mixed in the programmed ratio during the chromatographic run to effectively separate the components of the CCEs. The methanol content within the mobile phase mixture was set to 5%, 70%, 85%, and 5% at 0, 30, 40, and 55–60 min, respectively, after sample injection.

### 2.4. Cell Culture

A10 cells (#60127), which were VSMCs derived from the rat thoracic aorta, were obtained from the Bioresource Collection and Research Center (BCRC; Hsinchu, Taiwan). In addition, RAW264.7 cells (#60001), mouse BALB/c macrophages, were purchased from BCRC. All cells were cultured in Dulbecco's modified Eagle's medium (DMEM) that contained 10% fetal bovine serum (FBS), 1.5 g/L sodium bicarbonate, 4 mM L-glutamine, 4.5 g/L D-glucose, 100 U/L penicillin, and 100 mg/L streptomycin. In the culture medium of A10 cells, 1 mM sodium pyruvate was added. The cells were maintained at 37 °C in a humidified 5% CO_2_ incubator. The culture medium was refreshed every 2 days, and the cells were passaged 2-3 times weekly.

### 2.5. Cell Viability Analysis

The cells seeded on 96-well plates (8 × 10^3^ cells/well) were treated with CCEs for 24 h. Subsequently, the culture medium was replaced with fresh medium containing 0.5 mg/mL methylthialazole tetrazolium (MTT). After an additional 2 h of incubation, the medium was removed, and 100 *μ*L of dimethyl sulfoxide (DMSO) was added to dissolve formazan crystals. Cell viability was assessed by measuring the absorbance at 570 nm with a reference wavelength of 660 nm. The absorbance value of the control group (10% FBS alone) was defined as 100%.

### 2.6. Cell Migration Analysis (Transwell Assay)

Cell migration was analyzed using ThinCert cell culture inserts (#662638; Greiner Bio-One, Monroe, NC, USA). In brief, A10 cells seeded on the inner surface of the inserts (1 × 10^6^ cells/insert) mounted on a 24-well plate were treated with several concentrations of the CCEs under a serum-free (SF) condition, and the outside of the inserts was filled with normal culture medium. After 24 h of incubation, the inserts were removed from the culture plate, washed twice by using ice-cold 1 × phosphate-buffered saline, and then immersed in 10% formalin to fix the cells attached on the inserts. Subsequently, the cells located on the inner surface of the inserts were gently removed using a cotton swab. Thereafter, the cells attached on the lower surface of the inserts were stained with hematoxylin, and images were acquired using a Nikon Eclipse TS100 inverted microscope (Tokyo, Japan) equipped with a digital camera (#E3ISPM06300KPA, ToupTek Photonics, Hangzhou, Zhejiang, China). Cell numbers of each image were counted and averaged from five random fields. The degree of cell migration was calculated as the relative cell number to the group treated with 10% FBS alone.

### 2.7. Immunoblotting

Immunoblotting was performed following the procedure reported in our previous study with a minor modification [22]. In brief, harvested cells were lysed using the PRO-PREP Protein Extraction Solution with proteinase inhibitor cocktail (#4693159001, cOmplete, Roche) and phosphatase inhibitor cocktail (#04906837001, PhosSTOP, Roche). The cell lysates were centrifuged at 13000xg at 4°C for 10 min to collect supernatants. The protein concentrations of samples were measured using a BioRad protein assay kit (BioRad, Hercules, CA, USA), and a standard curve was developed using serial dilutions of bovine serum albumin. Subsequently, protein samples were electrophoresed using 10% sodium dodecyl sulfate-polyacrylamide gel electrophoresis and transferred onto polyvinylidene difluoride (PVDF) membranes (Immun-bot, BioRad). The blotted PVDF membrane was blocked with 5% nonfat dry milk in Tris-base saline containing 0.1% Tween 20 for 1 h, probed with a suitable primary antibody overnight at 4°C and incubated with the secondary antibody for 1 h at room temperature. Chemiluminescent signals were developed using a WesternBright ECL kit (#K-12045-D50, Advansta, Menlo Park, CA, USA) and then acquired using the Azure C300 imaging system (Azure Biosystem, Dublin, CA, USA). The band intensity was quantified using densitometric analysis software (AzureSpot version 14.0; Azure Biosystems) and then normalized to that of the internal control (GAPDH). Finally, the protein expression was calculated as the relative percentage to the group treated with 10% FBS alone.

### 2.8. Reverse Transcription-Polymerase Chain Reaction

Reverse transcription-polymerase chain reaction (PCR) was performed according to the procedure reported in a previous study with a minor modification. In brief, total RNA was extracted and then reverse transcribed into complementary (c) DNA by using 3-Zol reagent and a ReverTra Ace Set (#PU-TRT-200; TOYOBO, Osaka, Japan), respectively, according to the manufacturer's method prescribed in the manual. PCR reactions were conducted using the OnePCR HotStar system (#SM206-0100; GeneDireX, Miaoli, Taiwan) in a thermocycler (Labcycler; SensoQuest, Gottingen, Germany). The sequences of primer pairs were as follows: GAPDH (forward: 5′-ATCAAGAAGGTGGTGAAGCA-GGCG-3′ and reverse: 5′-GGGATGGAATTGTGAGGGAG-ATGCT C-3′), IL-1*β* (forward: 5′-TGCCACCTTTTGACA-GTG ATG-3′ and reverse: 5′-AAGGTCCACGGGAAAGA-CAC-3′), IL-6 (forward: 5′-GCCTTCTTGGGA CTGATG-CT-3′ and reverse: 5′-CTGCAAGTGCATCATCGTTGT-3′), and microRNA (miRNA)-132 (forward: 5′-GCAACCG-TGGCTT TCGAT T-3′ and reverse: 5′-CGACCATGGC-TGTAGACTGTTA-3′). PCR amplicons were verified on a 2% (w/v) agarose gel and visualized using a fluorescent dye (Novel Juice; GeneDireX, Taichung, Taiwan). Band signals were acquired and quantified using the Azure C300 imaging system and in AzureSpot software (Azure Biosystems), respectively. The band intensities of detected genes were normalized to that of the internal control (GAPDH). Finally, the gene expression was determined as the relative percentage to the group treated with 10% FBS alone.

### 2.9. Animal Model of Balloon Angioplasty

A total of 34 male Sprague-Dawley rats (weighing 250–300 g) purchased from BioLASCO (Taipei, Taiwan) were housed in a 12-h light/dark cycle and fed ad libitum in the Laboratory Animal Center of Taipei Medical University (TMU). The animal protocol was approved (#LAC-2017-0367) by the Institutional Animal Care and Use Committee and performed according to the institutional animal ethical guidelines of TMU. Rats were randomly divided into four groups as follows: group 1, sham control (normal control, NC); group 2, balloon injury (BI) + vehicle; group 3, BI + 8 *μ*g/mL CCE3; and group 4, BI + 16 *μ*g/mL CCE3. Different doses of CCE3 or vehicle were dissolved in 30% (w/v) Pluronic F-127 gels, a hydrogel-based polymer and drug carrier used for releasing the drug slowly. Neointimal hyperplasia was induced by an arterial embolectomy catheter (2 F × 80 cm; Biosensors International Technology, Singapore) in the left common carotid artery. Briefly, the animals were anesthetized by administering an intraperitoneal injection of 20 mg/kg Zoletil 50 (Virbac; Carros, France) and 5 mg/kg Rompun (Bayer; Mississauga, ON, Canada). Subsequently, an arterial embolectomy catheter was inserted into the left common carotid artery from an incision made in the external carotid artery. The catheter balloon was inflated under pressure (1.3 kg/cm^2^) and then pushed and pulled through the vascular lumen three times to damage the arterial wall. After sealing the incision made in the external carotid artery, the Pluronic F-127 gel containing CCE3 was directly applied on the outside of the common carotid artery damaged by balloon inflation and subsequent tissue suturing. Two weeks later, the rats were sacrificed, and their left carotid common arteries were harvested and cut into 10 *μ*m frozen sections by using a cryostat. The frozen sections were further stained with hematoxylin and eosin to observe the histopathological changes. The areas of vascular neointima and media layers were measured using an image analysis software, ImageJ (NIH, Bethesda, MD, USA). The ratio of the neointima-to-media area (N/M ratio) was used as a severity index of neointimal hyperplasia (restenosis).

### 2.10. Statistical Analysis

All variables are presented as their mean ± standard deviation. Differences among multiple groups were determined using one-way analysis of variance in combination with Dunnett's test. An unpaired *t*-test was performed to compare two independent groups. A *P* value of <0.05 was considered statistically significant.

## 3. Results

### 3.1. Total Extraction Yields and HPLC Analysis of CCEs

**Figure 1:**
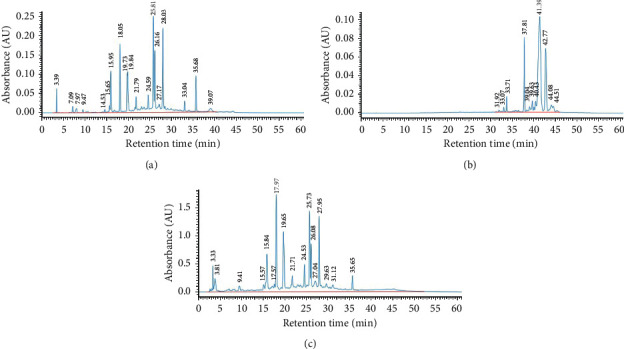
HPLC fingerprinting profiles of three different CCEs. (a) CCE1. (b) CCE2. (c) CCE3.

A total of 342 g of leaves and stems collected from *C. chinense* were used to prepare different CCEs. Three extracts (CC1, CC2, and CC3) of *C. chinense* were sequentially obtained using water, hexane, and ethanol extractions, respectively. The dry weights and total extraction yields of CCE1, CCE2, and CCE2 were 14.76 g (4.32%), 2.13 g (0.62%), and 20.44 g (5.98%), respectively. In addition, we developed the HPLC fingerprinting profiles of different CCEs to be used as references for monitoring the quality and batch reproducibility of the extraction procedure of each sample (Figure 1). On the basis of HPLC conditions used in the present study, CCE1 and CCE3 were found to have higher diversity and abundance in terms of their ingredients. Moreover, our analysis results verified that water and ethanolic extracts, namely, CCE1 and CCE3, respectively, had more polar compounds eluted rapidly at the early stage of separation, and ingredients with lower polarity were observed in the hexane extract CCE2.

### 3.2. CCE Inhibited Cell Viability and Migration in VSMCs

**Figure 2:**
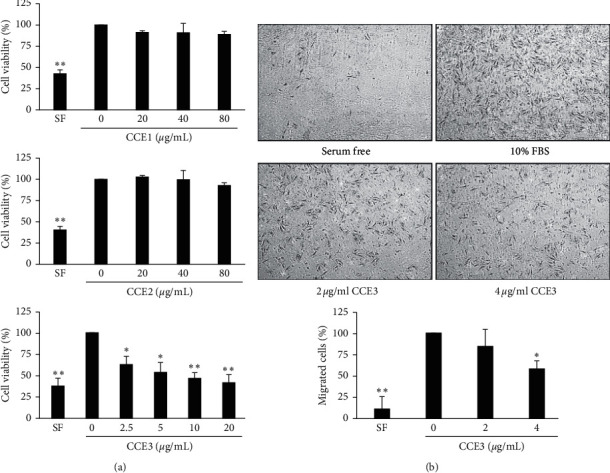
Inhibitory effects of CCE3 on VSMC growth and migration. Cell viability (a) and cell migration (b) were analyzed using MTT and transwell assays, respectively, in A10 cells treated with several concentrations of CCE3. ^*∗*^*P* < 0.05 and ^*∗∗*^*P* < 0.01 compared with the control group (10% FBS alone group).

To determine the CCE with the strongest potential to suppress VSMC viability, we examined the cytotoxicity of CCE1, CCE2, and CCE3 by using the MTT assay (Figure 2(a)). The results indicated that CCE3 exhibited the strongest activity (half maximal inhibitory concentration = 8 *μ*g/mL) for inhibiting 10% FBS-stimulated cell proliferation, and CCE1 and CCE2 exerted no significant effects on growth inhibition at the tested concentrations. Because growth suppression is a key effect of a candidate drug used for preventing neointimal formation, only CCE3 was selected for use in subsequent experiments.

To evaluate the antimigration effects of CCE3, the inhibitory effects of various doses (2 and 4 *μ*g/mL) of CCE3 on VSMC migration were determined using a transwell assay. The results revealed that 10% FBS markedly stimulated VSMC migration, a phenomenon that could be significantly inhibited after CCE3 treatment at a concentration of 4 *μ*g/mL (Figure 2(b)).

### 3.3. Regulation of Growth or Migration-Related Molecules in VSMCs by CCE3

**Figure 3:**
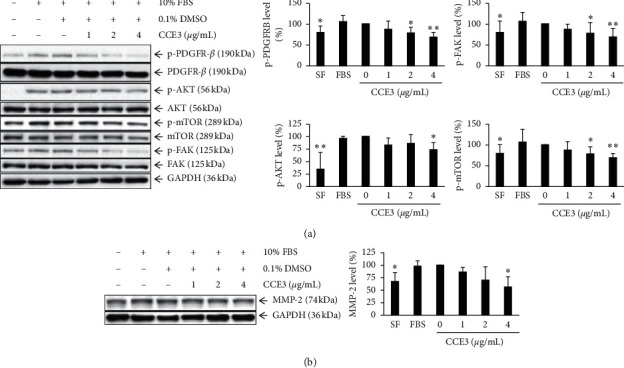
Regulation of growth- or migration-associated molecules by CCE3. A10 cells were starved for 24 h and then stimulated with 10% FBS for 30 min in the presence of various concentrations (1, 2, and 4 *μ*g/mL) of CCE3 to examine the phosphorylation levels of detected proteins (a) or to analyze the total levels of detected proteins after 24 h of incubation (b). ^*∗*^*P* < 0.05 and ^*∗∗*^*P* < 0.01 compared with the group treated with 10% FBS alone.

To elucidate the possible regulatory mechanisms underlying the role of CCE3 in VSMC proliferation and migration, we analyzed the activation or expression levels of several critical proteins through Western blotting. The experimental results indicated that CCE3 rapidly reduced the phosphorylated levels of PDGFRB and its downstream molecules (AKT and mTOR) in a dose-dependent manner (Figure 3(a)). Similarly, the activation of FAK was also suppressed after CCE3 treatment. After 24 h of incubation, the matrix metalloproteinase-2 (MMP2) protein was downregulated after CCE3 treatment (Figure 3(b)).

### 3.4. Regulation of Inflammation-Related Molecules in Macrophages and VSMCs by CCE3

**Figure 4:**
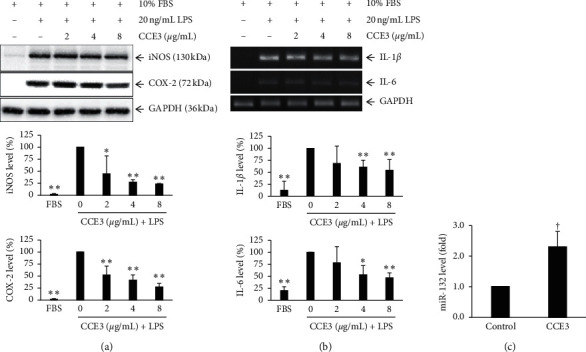
Regulation of LPS-induced inflammatory molecules by CCE3. RAW264.7 cells were incubated with several concentrations (2, 4, and 8 *μ*g/mL) of CCE3 in the presence of 20 ng/mL LPS to examine the expression levels of LPS-stimulated inflammatory enzymes at translation levels (a) and that of cytokines at transcriptional levels (b). The expression level of miRNA-132 was analyzed in A10 cells after 24 h of treatment with CCE3 (c). ^*∗*^*P* < 0.05 and ^*∗∗*^*P* < 0.01 relative to the group treated with LPS alone. ^†^*P* < 0.05 relative to the control group (10% FBS alone).

To evaluate whether CCE3 exerts anti-inflammatory effects, we induced several inflammation-associated molecules, namely, iNOS, COX-2, IL-1*β*, and IL-6, in RAW264.7 macrophages through stimulation with lipopolysaccharides (LPS; Figure 4). Our results demonstrated that LPS addition largely increased the translational levels of iNOS and COX-2 and that these levels were markedly inhibited after CCE3 treatment (Figure 4(a)). Similarly, LPS-stimulated increases in inflammatory cytokines, namely, IL-1*β* and IL-6, and significantly decreased in macrophages treated with CCE3 (Figure 4(b)). Moreover, our study results showed that the expression level of an anti-inflammation-related miRNA, miR-132, was significantly increased in VSMCs treated with CCE3 (Figure 4(c)).

### 3.5. CCE3 Inhibited BI-Induced Neointimal Formation

**Figure 5:**
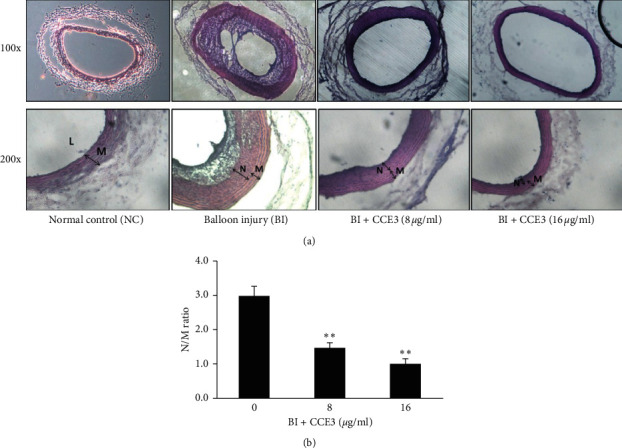
Preventive effect of CCE3 on balloon-induced neointima formation. Artery cross-sections were cut using a cryostat and stained with hematoxylin and eosin to examine histopathological changes in arteries by using a microscope at 100-fold (upper panel) and 200-fold magnification (lower panel), respectively (a). L, lumen; N, neointima; M, media. Severity of neointimal hyperplasia was quantified by determining the ratio of the neointima-to-media area (N/M ratio) (b). ^*∗∗*^*P* < 0.01 compared with the BI group.

To examine whether CCE3 can prevent neointima formation, a rat carotid injury model was established to induce neointimal hyperplasia (Figure 5). Two weeks after surgery, the injured carotid arteries were harvested, cut as frozen sections, and then stained with hematoxylin and eosin to observe histopathological changes in the different layers of the arterial wall (Figure 5(a)). The severity of (neo) intimal hyperplasia was determined by calculating the ratio of the neointima-to-media area (N/M ratio), and our findings indicated that the N/M ratio was increased in the BI group than in the sham (normal) control group (Figure 5(b)). By contrast, the N/M ratio of the BI group treated with CCE3 was markedly decreased when compared with that of the BI group.

## 4. Discussion

PDGF, a dimeric molecule, is classified into three isoforms (PDGF-AA, PDGF-AB, and PDGF-BB) based on the different assemblies of two polypeptide chains (A and B chains). PDGF plays critical pathological roles by regulating VSMCs and is involved in the progression of neointimal hyperplasia. Among the three isoforms, PDGF-BB exhibited the highest potential to stimulate VSMC growth [23]. PDGF-B and PDGFRB were found to be increased in injured arteries after percutaneous transluminal coronary angioplasty [24]. The PI3K-AKT-mTOR pathway, a crucial downstream cascade of PDGFRs, participated in PDGF-mediated physiological and pathological regulation [25, 26]. Inhibiting PDGFRs or targeting the PI3K-AKT pathway could alleviate excessive neointimal hyperplasia [27, 28]. MMP2, a gelatinase that can digest the extracellular matrix, was increased in the balloon-injured arteries of the hypercholesterolemic rabbit [29], and neutralizing MMP2 activity could inhibit VSMC migration [30]. Seo et al. indicated that MMP2 production can be increased in VSMCs through PDGFRB-mediated AKT activation [31]. In addition to MMP2, FAK is a molecule involved in regulating cell migration, and suppressing FAK can inhibit glucose-induced VSMC migration [32]. In the present study, we found that CCE3 reduced the activations of FAK and PDGFRB and attenuated the PDGFRB-mediated downstream PI3K-AKT-mTOR signaling pathway in VSMCs (Figure 3(a)). In addition, the expression of MMP2 was suppressed after CCE3 treatment (Figure 3(b)).

Gelatinases (MMP2 and MMP9) are secreting proteases that can digest the extracellular matrix (gelatin). The abnormal expression and activation of gelatinases have been linked to various pathological conditions such as atherosclerosis, tumor metastasis, and chronic obstructive pulmonary disease. In a balloon-injured rat carotid artery model, both gelatinases were found to be increased and activated in neointima lesions [33, 34], and either silencing MMP2 or MMP9 could effectively inhibit endovascular injury-induced neointimal growth [35]. The partial inhibitory mechanisms underlying the prevention of neointimal formation may be attributed to the gelatinase deficiency-caused reduction in the invasion, migration, and proliferation of VSMCs [36, 37]. In addition, the use of a drug-eluting stent coated with a broad-spectrum MMP inhibitor (GM6001) could prevent the development of restenosis [38]. The aforementioned findings imply that suppressing MMP expression or activity can be an effective strategy for preventing neointimal formation. The decrease in the translational level of MMP2 observed in the present study might contribute to the inhibitory effect of CCE3 on neointimal formation (Figure 3(b)).

The extracellular matrix (ECM) provides structural and biochemical support for resident cells and regulates many cellular functions such as proliferation, migration, adhesion, and differentiation. ECM remodeling not only plays a critical role in physiological processes, such as wound healing, but also participates in several pathological conditions, such as tumor metastasis and tissue fibrosis. Collagen and elastin, two major ECM components present within the vessel wall, maintain the structural integrity and elasticity of the vascular wall. Thus, alterations in the expression levels or ratios of these matrix proteins can lead to the occurrence of vascular remodeling. In the rat carotid artery BI model, a histopathological analysis showed that the synthesis and content of collagen and elastin were significantly increased in neointimal lesions [39, 40]. Nuthakki et al. found that lysyl oxidase, an enzyme involved in the cross-linking and stabilization of collagen and elastin in the vascular wall, was increased within the injured carotid arteries [41]. The increase in the contents of collagen and elastin in injured carotid arteries might be attributed to the increase in lysyl oxidase within these arteries [39, 40].

PDGF can induce COX-2 expression in VSMCs, and this effect can be suppressed by a PDGFR blocker [42]. COX-2 is an enzyme involved in prostaglandin biosynthesis and is a common target of nonsteroidal anti-inflammatory drugs, a type of anti-inflammatory and analgesic drug. COX-2-derived prostaglandin E2 facilitated the development of neointimal hyperplasia, and COX-2 knockout mice exhibited protection against wire injury-induced neointima formation [43]. In addition to COX-2, iNOS is a potential target induced by PDGF [44]. Nitric oxide (NO) is involved in critical physiological functions, such as in the vascular tone and immune response of phagocytes. However, NO is a free radical that might damage crucial biological components such as protein and DNA. Thus, excessive NO production induced by iNOS can damage tissues by generating oxidative stress, resulting in diseases. An in vivo study demonstrated that the knockdown of iNOS could attenuate neointima formation after perivascular arterial injury [45]. In the present study, two inflammation-associated enzymes, namely, iNOS and COX-2 were found to be decreased in VSMCs after CCE3 treatment (Figure 4(a)).

Several inflammatory cytokines, such as IL-1*β* and IL-6, cause the accumulation of leukocytes and inflammatory cells, modulate the proliferation of VSMCs, and are involved in formation of neointimal hyperplasia. An animal study suggested that the knockdown of IL-1 receptor (IL-1R) or the use of IL-1R antagonists could significantly reduce the N/M ratio in balloon-injured arteries [46]. In addition, IL-1*β* participates in the progression of restenosis by promoting MCP-1 expression in VSMCs [47]. IL-6 was upregulated by PDGF stimulation in VSMCs [48]. IL-6 can result in neointima formation by mobilizing bone marrow-derived cells to the vascular wall [49]. Furthermore, IL-6 can participate in VSMC migration and neointima formation induced by 15(S)-hydroxyeicosatetraenoic acid, a metabolite of arachidonic acid [50]. Our findings indicated that the transcriptional levels of IL-1*β* and IL-6 were decreased in VSMCs treated with CCE3 (Figure 4(b)).

MicroRNAs (miRNA) are noncoding RNAs that regulate posttranscriptional gene expression to alter the cellular functions of differentiation, proliferation, and apoptosis. In a previous study, miRNA-132 was found to inhibit VSMC hyperplasia and intima hypertrophy by suppressing the gene expression of leucine-rich repeat flightless-interacting protein 1 (Lrrfip1) [51]. In addition, a study reported the potential anti-inflammatory function of miRNA-132 in macrophages [52]. MiRNA-132 can directly target or indirectly modulate inflammation-associated molecules, such as COX-2, IL-1*β*, and IL-6, by suppressing the expression of p300 protein, thus reducing the expression of these molecules [53, 54]. In the present study, miRNA-132 was found to be significantly increased after CCE3 treatment. The effect of miRNA-132 might contribute to the prevention of inflammation and neointima formation (Figure 4(c)).

Chang et al. indicated that compared with methanolic extract, the aqueous extract of *C. chinense* exhibited a stronger antiproliferative activity in human HepG2 cells [4]. In our study, we observed that CCE3, an alcoholic extract, significantly suppressed VSMC growth compared with the aqueous extract (CCE1). The differences between the findings of our study and those of previous studies may be due to the following reasons. First, CCE3 was obtained from the residue left after sequential extractions through the use of aqueous and n-hexane; thus, the chemical compositions of ethanolic extracts may vary between our study and previous studies. Second, different types (normal vs. malignant cells and hepatocytes vs. VSMCs) of cells may have diverse susceptibility to the same treatment. Third, extracts from different plant parts (e.g., leaf, stem, root, flower, or whole plant) may have different chemical constituents and activities. In addition to crude extracts, numerous compounds of *C. chinense* were found to exhibit various biological effects including antiproliferation, antimigration, anti-inflammation, and antithrombosis. Hispidulin, taraxerol, and taraxerol acetate can suppress the cell growth of human cancer cells through various pharmacological mechanisms including cell-cycle arrest, apoptosis, and autophagy [18, 19, 55]. Similarly, betulin, lupeol, and oleanolic acid have demonstrated cytotoxicity against cancer cells [56–58]. Among these compounds, taraxerol acetate was found to exhibit antimigration activity. Scopoletin and taraxerol acetate suppressed inflammation in *λ*-carrageenan-stimulated paw edema, and these effects may be partially attributed to decreases in iNOS and COX-2 expression in the edema paw and to the reduction in the serum levels of nitric oxide, tumor necrosis factor-*α*, and prostaglandin E2 [59, 60]. In addition, hispidulin exhibited the anti-inflammatory activity by reducing NF-*κ*b-dependent transcription in a 12-O-tetradecanoylphorbol-13-acetate-induced mouse ear edema model [61] and exerted an inhibitory effect, thus preventing platelet aggregation by increasing the cAMP level [62]. Bioactivities of the aforementioned compounds provide some clues to partially explain the preventive activity of CCE3 that operates by suppressing inflammation, VSMC proliferation and migration, and subsequent neointima formation (Figures 2 and 5).

The abnormal expression profiles of some molecules, such as cathepsins, play pathological roles in the development of cardiovascular diseases such as atherosclerosis and restenosis. Cathepsins are a group of lysosomal cysteine proteases involved in various physiological processes such as apoptosis, antigen processing, and extracellular matrix remodeling. A previous study showed that both cathepsin S and K were upregulated in the neointima lesions of rat balloon-injured arteries, thus contributing to pathological arterial remodeling [63]. Similarly, suppressing cathepsin K attenuated flow cessation-induced intimal hyperplasia in ApoE^−/−^ mice [64]. Thus, pharmacological interventions targeting cathepsin S or cathepsin K may be a possible strategy for the treatment of injury-related vascular remodeling and neointimal hyperplasia.

## 5. Conclusions

**Figure 6:**
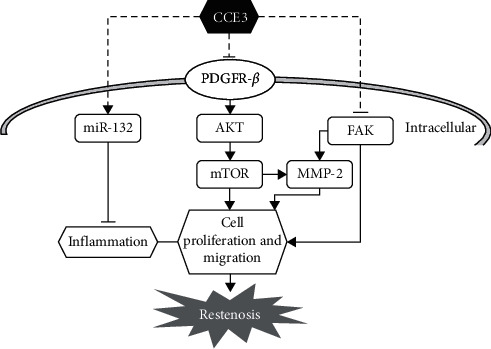
Schematic overview of the pharmacological mechanism of CCE3 in the prevention of neointimal hyperplasia.

Our study findings suggest that CCE3, the ethanolic extract of *C. chinense*, could inhibit VMSC proliferation and migration and prevent neointima formation. CCE3 may exhibit these biological activities by suppressing the activation of the PDGFRB-mediated PI3K-AKT-mTOR pathway, inhibiting the expression of inflammation-associated molecules, namely, iNOS, COX-2, IL-1*β*, and IL-6, increasing the level of anti-inflammatory and antirestenosis-related miRNA, miR-132, and reducing the expression of cell migration-associated molecules, FAK and MMP2 (Figure 6).

## Data Availability

The data used to support the findings of this study are available from the corresponding author upon request.

## References

[1] Popma J. J., Califf R. M., Topol E. J. (1991). Clinical trials of restenosis after coronary angioplasty. *Circulation*.

[2] Kuntz R. E., Baim D. S. (2000). Prevention of coronary restenosis. *Circulation*.

[3] Curcio A., Torella D., Indolfi C. (2011). Mechanisms of smooth muscle cell proliferation and endothelial regeneration after vascular injury and stenting: approach to therapy. *Circulation Journal*.

[4] Chang T. N., Huang G. J., Ho Y. L. (2009). Antioxidant and antiproliferative activities of *Crossostephium chinensis* (L.) Makino. *American Journal of Chinese Medicine*.

[5] Raines E. W., Bowen-Pope D. F., Ross R., Sporn M. B., Roberts A. B. (1990). Platelet-derived growth factor. *Peptide Growth Factors and Their Receptors I, Handbook of Experimental Pharmacology*.

[6] Ross R., Glomset J., Kariya B., Harker L. (1974). A platelet-dependent serum factor that stimulates the proliferation of arterial smooth muscle cells in vitro. *Proceedings of the National Academy of Sciences*.

[7] Jawien A., Bowen-Pope D. F., Lindner V., Schwartz S. M., Clowes A. W. (1992). Platelet-derived growth factor promotes smooth muscle migration and intimal thickening in a rat model of balloon angioplasty. *Journal of Clinical Investigation*.

[8] Ferns G., Raines E., Sprugel K., Motani A., Reidy M., Ross R. (1991). Inhibition of neointimal smooth muscle accumulation after angioplasty by an antibody to PDGF. *Science*.

[9] Bilder G., Wentz T., Leadley R. (1999). Restenosis following angioplasty in the swine coronary artery is inhibited by an orally active PDGF-receptor tyrosine kinase inhibitor, RPR101511A. *Circulation*.

[10] Jandt E., Mutschke O., Mahboobi S. (2010). Stent-based release of a selective PDGF-receptor blocker from the bis-indolylmethanon class inhibits restenosis in the rabbit animal model. *Vascular Pharmacology*.

[11] Rollins B. J., Morrison E. D., Stiles C. D. (1988). Cloning and expression of JE, a gene inducible by platelet-derived growth factor and whose product has cytokine-like properties. *Proceedings of the National Academy of Sciences*.

[12] Miller M. D., Krangel M. S. (1992). Biology and biochemistry of the chemokines: a family of chemotactic and inflammatory cytokines. *Critical Reviews in Immunology*.

[13] Eun S. Y., Ko Y. S., Park S. W., Chang K. C., Kim H. J. (2015). IL-1*β* enhances vascular smooth muscle cell proliferation and migration via P2Y2 receptor-mediated RAGE expression and HMGB1 release. *Vascular Pharmacology*.

[14] Rectenwald J. E., Moldawer L. L., Huber T. S., Seeger J. M., Ozaki C. K. (2000). Direct evidence for cytokine involvement in neointimal hyperplasia. *Circulation*.

[15] Grassia G., Maddaluno M., Guglielmotti A. (2009). The anti-inflammatory agent bindarit inhibits neointima formation in both rats and hyperlipidaemic mice. *Cardiovascular Research*.

[16] Che Man R., Sulaiman N., Ishak M. F. (2020). The effects of pro-inflammatory and anti-inflammatory agents for the suppression of intimal hyperplasia: an evidence-based review. *International Journal of Environmental Research and Public Health*.

[17] Chang Y. S., Lin I. H. (2003). *Compendium of Medicinal Plants Used by the Indigenous People of Taiwan*.

[18] Tan B., Shi H. L., Ji G., Xie J. Q. (2011). Effects of taraxerol and taraxeryl acetate on cell cycle and apoptosis of human gastric epithelial cell line AGS. *Journal of Chinese Integrative Medicine*.

[19] Hong J.-F., Song Y.-F., Liu Z., Zheng Z.-C., Chen H.-J., Wang S.-S. (2016). Anticancer activity of taraxerol acetate in human glioblastoma cells and a mouse xenograft model via induction of autophagy and apoptotic cell death, cell cycle arrest and inhibition of cell migration. *Molecular Medicine Reports*.

[20] Akihisa T., Yasukawa K., Oinuma H. (1996). Triterpene alcohols from the flowers of compositae and their anti-inflammatory effects. *Phytochemistry*.

[21] Pan C.-H., Hsieh I.-C., Liu F.-C. (2010). Effects of a Chinese herbal health formula, “Gan-Lu-Yin”, on angiogenesis. *Journal of Agricultural and Food Chemistry*.

[22] Pan C. H., Chen S. Y., Wang J. Y. (2020). Sclareol ameliorated ERCC1-mediated cisplatin resistance in A549 human lung adenocarcinoma cells and a murine xenograft tumor model by suppressing AKT-GSK3beta-AP1/Snail and JNK-AP1 pathways. *Chemico-Biological Interactions*.

[23] Kondo T., Konishi F., Inui H., Inagami T. (1993). Differing signal transductions elicited by three isoforms of platelet-derived growth factor in vascular smooth muscle cells. *Journal of Biological Chemistry*.

[24] Tanizawa S., Ueda M., van der Loos C. M., van der Wal A. C., Becker A. E. (1996). Expression of platelet derived growth factor B chain and beta receptor in human coronary arteries after percutaneous transluminal coronary angioplasty: an immunohistochemical study. *Heart*.

[25] Zhang H., Bajraszewski N., Wu E. (2007). PDGFRs are critical for PI3K/Akt activation and negatively regulated by mTOR. *Journal of Clinical Investigation*.

[26] Xiao Y., Peng H., Hong C. (2017). PDGF promotes the Warburg effect in pulmonary arterial smooth muscle cells via activation of the PI3K/AKT/mTOR/HIF-1*α* signaling pathway. *Cellular Physiology and Biochemistry*.

[27] Tang F., Liu M., Zeng O. (2019). Gefitinib-coated balloon inhibits the excessive hyperplasia of intima after vascular injuries through PI3K/AKT pathway. *Technology and Health Care*.

[28] Levitzki A. (2005). PDGF receptor kinase inhibitors for the treatment of restenosis. *Cardiovascular Research*.

[29] Feldman L. J., Mazighi M., Scheuble A. (2001). Differential expression of matrix metalloproteinases after stent implantation and balloon angioplasty in the hypercholesterolemic rabbit. *Circulation*.

[30] Pauly R. R., Passaniti A., Bilato C. (1994). Migration of cultured vascular smooth muscle cells through a basement membrane barrier requires type IV collagenase activity and is inhibited by cellular differentiation. *Circulation Research*.

[31] Seo K. W., Lee S. J., Kim Y. H. (2013). Mechanical stretch increases MMP-2 production in vascular smooth muscle cells via activation of PDGFR-beta/Akt signaling pathway. *PLoS One*.

[32] Lin Y. C., Chen L. H., Varadharajan T. (2014). Resveratrol inhibits glucose‐induced migration of vascular smooth muscle cells mediated by focal adhesion kinase. *Molecular Nutrition & Food Research*.

[33] Jenkins G. M., Crow M. T., Bilato C. (1998). Increased expression of membrane-type matrix metalloproteinase and preferential localization of matrix metalloproteinase-2 to the neointima of balloon-injured rat carotid arteries. *Circulation*.

[34] Webb K. E., Henney A. M., Anglin S., Humphries S. E., McEwan J. R. (1997). Expression of matrix metalloproteinases and their inhibitor TIMP-1 in the rat carotid artery after balloon injury. *Arteriosclerosis, Thrombosis, and Vascular Biology*.

[35] Guo L., Ning W., Tan Z., Gong Z., Li X. (2014). Mechanism of matrix metalloproteinase axis-induced neointimal growth. *Journal of Molecular and Cellular Cardiology*.

[36] Kuzuya M., Kanda S., Sasaki T. (2003). Deficiency of gelatinase a suppresses smooth muscle cell invasion and development of experimental intimal hyperplasia. *Circulation*.

[37] Cho A., Reidy M. A. (2002). Matrix metalloproteinase-9 is necessary for the regulation of smooth muscle cell replication and migration after arterial injury. *Circulation Research*.

[38] Song J. B., Shen J., Fan J. (2020). Effects of a matrix metalloproteinase inhibitor-eluting stent on in-stent restenosis. *Medical Science Monitor*.

[39] Nili N., Zhang M., Strauss B. H., Bendeck M. P. (2002). Biochemical analysis of collagen and elastin synthesis in the balloon injured rat carotid artery. *Cardiovascular Pathology*.

[40] Thyberg J., Blomgren K., Hedin U., Dryjski M. (1995). Phenotypic modulation of smooth muscle cells during the formation of neointimal thickenings in the rat carotid artery after balloon injury: an electron-microscopic and stereological study. *Cell and Tissue Research*.

[41] Nuthakki V. K., Fleser P. S., Malinzak L. E. (2004). Lysyl oxidase expression in a rat model of arterial balloon injury. *Journal of Vascular Surgery*.

[42] Xu K., Kitchen C. M., Shu H.-K. G., Murphy T. J. (2007). Platelet-derived growth factor-induced stabilization of cyclooxygenase 2 mRNA in rat smooth muscle cells requires the c-Src family of protein-tyrosine kinases. *Journal of Biological Chemistry*.

[43] Zhang J., Zou F., Tang J. (2013). Cyclooxygenase-2-Derived prostaglandin E 2 promotes injury-induced vascular neointimal hyperplasia through the E-prostanoid 3 receptor. *Circulation Research*.

[44] Kunz D., Walker G., Eberhardt W., Messmer U. K., Huwiler A., Pfeilschifter J. (1997). Platelet-derived growth factor and fibroblast growth factor differentially regulate interleukin 1beta- and cAMP-induced nitric oxide synthase expression in rat renal mesangial cells. *Journal of Clinical Investigation*.

[45] Chyu K.-Y., Dimayuga P., Zhu J. (1999). Decreased neointimal thickening after arterial wall injury in inducible nitric oxide synthase knockout mice. *Circulation Research*.

[46] Chamberlain J., Evans D., King A. (2006). Interleukin-1*β* and signaling of interleukin-1 in vascular wall and circulating cells modulates the extent of neointima formation in mice. *The American Journal of Pathology*.

[47] Lim J. H., Um H. J., Park J.-W., Lee I.-K., Kwon T. K. (2009). Interleukin-1*β* promotes the expression of monocyte chemoattractant protein-1 in human aorta smooth muscle cells via multiple signaling pathways. *Experimental and Molecular Medicine*.

[48] Loppnow H., Libby P. (1990). Proliferating or interleukin 1-activated human vascular smooth muscle cells secrete copious interleukin 6. *Journal of Clinical Investigation*.

[49] Shoji M., Furuyama F., Yokota Y. (2014). IL-6 mobilizes bone marrow-derived cells to the vascular wall, resulting in neointima formation via inflammatory effects. *Journal of Atherosclerosis and Thrombosis*.

[50] Chava K. R., Karpurapu M., Wang D. (2009). CREB-mediated IL-6 expression is required for 15(S)-hydroxyeicosatetraenoic acid-induced vascular smooth muscle cell migration. *Arteriosclerosis, Thrombosis, and Vascular Biology*.

[51] Choe N., Kwon J.-S., Kim J.-R. (2013). The microRNA miR-132 targets Lrrfip1 to block vascular smooth muscle cell proliferation and neointimal hyperplasia. *Atherosclerosis*.

[52] Liu F., Li Y., Jiang R. (2015). miR-132 inhibits lipopolysaccharide-induced inflammation in alveolar macrophages by the cholinergic anti-inflammatory pathway. *Experimental Lung Research*.

[53] van Zonneveld A. J., Au Y. W., Stam W. (2020). MicroRNA-132 regulates salt-dependent steady-state renin levels in mice. *Communications Biology*.

[54] Lagos D., Pollara G., Henderson S. (2010). miR-132 regulates antiviral innate immunity through suppression of the p300 transcriptional co-activator. *Nature Cell Biology*.

[55] Yu C. Y., Su K. Y., Lee P. L. (2013). Potential therapeutic role of hispidulin in gastric cancer through induction of apoptosis via NAG-1 signaling. *Evidence-Based Complementary and Alternative Medicine*.

[56] Sultana N., Ata A. (2008). Oleanolic acid and related derivatives as medicinally important compounds. *Journal of Enzyme Inhibition and Medicinal Chemistry*.

[57] Drag M., Surowiak P., Drag-Zalesinska M., Dietel M., Lage H., Oleksyszyn J. (2009). Comparision of the cytotoxic effects of birch bark extract, betulin and betulinic acid towards human gastric carcinoma and pancreatic carcinoma drug-sensitive and drug-resistant cell lines. *Molecules*.

[58] Saleem M. (2009). Lupeol, a novel anti-inflammatory and anti-cancer dietary triterpene. *Cancer Letters*.

[59] Chang T. N., Deng J. S., Chang Y. C. (2012). Ameliorative effects of Scopoletin from *Crossostephium chinensis* against inflammation pain and its mechanisms in mice. *Evidence-Based Complementary and Alternative Medicine*.

[60] Rahman U. u., Ali S., Ubaidullah, Khan I. (2016). Anti-inflammatory activity of taraxerol acetate. *Journal of Medical Sciences*.

[61] Clavin M., Gorzalczany S., Macho A. (2007). Anti-inflammatory activity of flavonoids from *Eupatorium arnottianum*. *Journal of Ethnopharmacology*.

[62] Bourdillat B., Delautier D., Labat C., Benveniste J., Potier P., Brink C. (1988). Hispidulin, a natural flavone, inhibits human platelet aggregation by increasing cAMP levels. *European Journal of Pharmacology*.

[63] Cheng X. W., Kuzuya M., Sasaki T. (2004). Increased expression of elastolytic cysteine proteases, cathepsins S and K, in the neointima of balloon-injured rat carotid arteries. *The American Journal of Pathology*.

[64] Donners M. M., Bai L., Lutgens S. P. (2016). Cathepsin K deficiency prevents the aggravated vascular remodeling response to flow cessation in ApoE-/- mice. *PLoS One*.

